# Prospects and Challenges of Electrospun Cell and Drug Delivery Vehicles to Correct Urethral Stricture

**DOI:** 10.3390/ijms231810519

**Published:** 2022-09-10

**Authors:** Saeed Farzamfar, Elissa Elia, Stéphane Chabaud, Mohammad Naji, Stéphane Bolduc

**Affiliations:** 1 Centre de Recherche en Organogénèse Expérimentale/LOEX, Regenerative Medicine Division, CHU de Québec-Université Laval Research Center, Québec, QC G1J 1Z4, Canada; 2 Urology and Nephrology Research Center, Shahid Beheshti University of Medical Sciences, Tehran 1666677951, Iran; 3 Department of Surgery, Faculty of Medicine, Laval University, Québec, QC G1V 0A6, Canada

**Keywords:** regenerative medicine, tissue engineering, electrospinning, urethra, drug delivery, cell delivery

## Abstract

Current therapeutic modalities to treat urethral strictures are associated with several challenges and shortcomings. Therefore, significant strides have been made to develop strategies with minimal side effects and the highest therapeutic potential. In this framework, electrospun scaffolds incorporated with various cells or bioactive agents have provided promising vistas to repair urethral defects. Due to the biomimetic nature of these constructs, they can efficiently mimic the native cells’ niches and provide essential microenvironmental cues for the safe transplantation of multiple cell types. Furthermore, these scaffolds are versatile platforms for delivering various drug molecules, growth factors, and nucleic acids. This review discusses the recent progress, applications, and challenges of electrospun scaffolds to deliver cells or bioactive agents during the urethral defect repair process. First, the current status of electrospinning in urethral tissue engineering is presented. Then, the principles of electrospinning in drug and cell delivery applications are reviewed. Finally, the recent preclinical studies are summarized and the current challenges are discussed.

## 1. Introduction

Urethral stricture (US) is characterized by fibrosis of the tissues surrounding the urethral lumen that restricts the easy flow of fluids. While US is rare in females, it mainly affects the male population. In this regard, it is estimated that the incidence of US in males is about 1% after age 55 [1]. Urethral infections, overactive bladder, and traumatic injuries are the leading causes of US in females; while, iatrogenic damages, idiopathic, traumatic, and inflammatory events are the primary causes of US in males [2,3]. 

US commonly manifests with other symptoms such as urinary tract infection, urolithiasis, chronic inflammation, development of fistulas, and kidney injuries [4,5]. Therefore, there is an urgent need to treat US; otherwise, it may lead to renal failure and a significant reduction in the patients’ quality of life. 

The success of the current treatment modalities is determined by the size and location of the injury. US management strategies may vary from urethral dilatation and urethrotomy to autologous tissue grafting and anastomotic urethroplasty [6,7]. In critically sized urethral injuries, autologous tissues from various sources such as penile skin, oral mucosa, bladder mucosa, and intestinal mucosa are used to bridge the defect site. However, these tissues are not specialized to endure urine exposure, and their application may lead to additional complications such as infection, fistula development, malignancy, graft failure, renal failure, and recurrence of US [8,9]. Therefore, significant strides have been made to develop alternative strategies. In this framework, tissue engineering has shed new light on the next generation of urethral grafts. This group of technologies aims to develop artificial urethra by integrating biomimetic scaffolds, cell-based therapies, advanced drug delivery systems, and signaling molecules [10,11]. To this end, numerous studies have combined the principles of cell-based therapies, drug delivery, and tissue engineering to develop potential treatment modalities for US [12,13].

Indeed, biomimetic tissue-engineered scaffolds are ideal candidates for US treatment, as they can deliver various therapeutics such as cells and drugs. These constructs can be engineered to target specific pathophysiological processes involved in US development, induce tissue repair, trigger extracellular matrix secretion, and prevent the recurrence of US [1,14]. Selecting an appropriate fabrication method is primarily important to engineering such a scaffold. Among various candidates, electrospinning has gained significant therapeutic appeal. Based on biomimetic principles, electrospun scaffolds are highly similar to architectural features of native tissue extracellular matrix (ECM) and can build a permissive environment for cellular colonization, differentiation, and tissue remodeling [15,16]. Furthermore, electrospun scaffolds are ideal for developing drug-delivering urethral grafts due to their high surface-to-volume ratio, high encapsulation efficacy, and controlled drug release [17,18]. Excellent tensile strength, suturability, ease of fabrication, and tunable degradation rate are other therapeutic footholds of electrospun scaffolds in urethral defects repair [8]. 

This review discusses the recent progress, applications, and challenges of electrospun scaffolds to deliver cells or bioactive agents during urethral defects repair. First, the current status of electrospinning in urethra tissue engineering is presented. Then, the principles of electrospinning in drug and cell delivery applications are reviewed. Finally, the recent preclinical studies are summarized and the current challenges are discussed. 

## 2. Pathophysiology of US, Its Etiology, and Current Treatment Options

**Figure 1:**
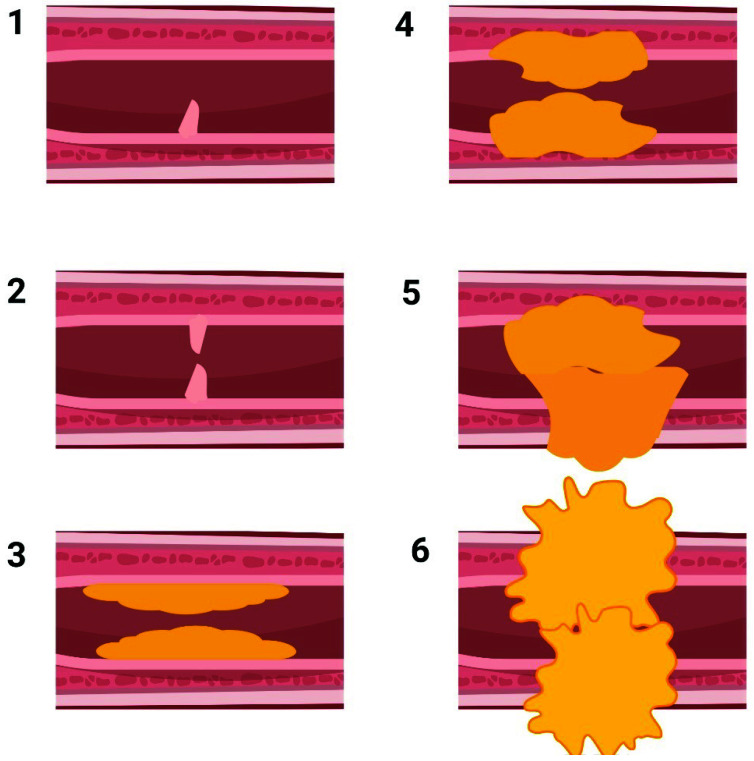
Schematic illustration representing the pathophysiology of urethra stricture. Stage 1 shows mucosal fold. In this stage, minor defects in the metaplastic tissue cause urinary extravasation that triggers the establishment of a fibrotic reaction. Increased deposition of ECM components results in the progression to further stages of US. Stage 2, shows Iris constriction. In stage 3, the fibrotic reaction has penetrated the spongiosum and caused a minimal fibrosis in the spongy tissue. Stage 4 shows full-thickness spongiofibrosis. In stage 5, fibrosis has penetrated the tissues outside the corpus spongiosum. In stage 6, a complex US has formed that is usually accompanied by a fistula.

The urethra lumen is lined with a pseudo-stratified columnar epithelium consisting of several layers of epithelial cells supported by a basement membrane [19]. Generally, all damages to the urethra epithelium or corpus spongiosum may potentially cause US. After the injury, where the inherent healing activity of epithelium fails to replace the lost cells, this layer is substituted with squamous metaplasia [20,21]. Small defects in the metaplastic tissue result in urine extravasation that subsequently causes the infiltration of polymorphonuclear cells to the injury site. These cells and the myofibroblast cells trigger a fibrotic reaction that finally results in the US [22,23]. In this process, the normal connective tissue of the urethra dramatically changes, and the ratio of type III to type I collagen decreases. Furthermore, smooth muscle cells are gradually replaced with dense collagen fibers, and the elasticity of the whole construct is compromised (Figure 1) [1,24]. 

The anterior US commonly occurs after traumatic injuries or infections in which the injured parts of the corpus spongiosum undergo fibrosis and cause the narrowing of the urethral lumen. In contrast, stenosis in the posterior urethra is not generally classified as true strictures. In this condition, iatrogenic causes or traumatic injuries, such as pelvic fractures, radical prostatectomy, and pelvic radiation cause a fibrotic reaction that narrows the lumen of the posterior urethra [6,25]. 

The etiology of US may involve various factors such as idiopathic causes, iatrogenic injuries, traumatic events, infectious diseases, and Lichen sclerosus [26,27]. However, there are still cases with unknown etiology. While in western countries, the iatrogenic causes account for the majority of disease etiology, US in developing countries is primarily caused by infectious diseases and traumatic injuries [28,29]. Lichen sclerosus is a chronic inflammatory response of the skin with a predilection for the genital region, that can cause obstructive urethral scarring [30].

Different treatment options have been developed to alleviate US. In this regard, urethral dilation with various instruments such as balloons, sounds, and catheters has been used to increase the caliber of obstructed urethra [31]. However, this treatment strategy stretches the urethra’s wall and may introduce some defects. Therefore, urinary extravasation is possible after urethral dilation, explaining the high recurrence rate of US following this treatment. In addition, this treatment is not curative, as it does not cure the fibrotic lesion but only corrects its symptoms, leaving in place the mechanisms underlying the appearance of the lesion. Therefore, at best, it can only be considered a temporary treatment allowing the surgeon to implement a complete strategy [32,33]. 

With the advent of direct visual internal urethrotomy (DVIU), the use of blind urethra dilation has been considerably limited. In this technique, a cold-knife longitudinal incision at the stricture site is made to release the scar tissue under the surgeon’s direct vision using a cystoscope [34,35]. Besides cold-knife, various lasers have been widely used for internal urethrotomy [36]. Generally, the success rate of this procedure is determined by the size and location of the injury. Risk factors for recurrent US are the previous attempts at urethrotomy, large-sized urethral defects (>2 cm), infection, US at the penile or membranous urethra, and strictures at different locations. The shortcomings of this technique include hemorrhage, recurrent US, urine extravasation, and perineal hematoma [37,38].

Application of temporary or permanent stents after dilation or internal urethrotomy has also been proposed. However, their various complications, such as infection, high rate of lumen obstruction, stent movement, and chronic pain, have led to their low success rate [39,40].

The success rate of reconstructive urethroplasty is much higher than other existing strategies. Therefore, urethroplasty is considered the gold standard of US treatment. In this framework, various methods such as end-to-end cooptation (anastomosis), onlay grafting, and the use of flaps have been developed [14,41]. The end-to-end anastomosis technique is often utilized to treat injuries less than 2 cm [42]. On the other hand, the graft urethroplasty technique is used to treat injuries >2 cm, as the end-to-end anastomosis will not be tension-free in these cases. In this method, tissue grafts from various sources such as the skin, the bladder, the oral mucosa, and the intestinal mucosa are harvested to bridge the defect site [43,44]. However, these tissues are not specialized for urine exposure. As a result, complications such as infection, fistula development, malignancy, graft failure, renal failure, and US recurrence may arise [8,14].

As shown, the current treatment options are minimal, with numerous drawbacks. Fortunately, regenerative medicine has broken new grounds to address this dilemma. 

## 3. Pros and Cons of Potential Cell Sources to Repair Urethra Defects

Cells from different lineages are available to repair urethral defects. In this context, several cell types including autologous urothelial cells, smooth muscle cells, cells derived from buccal mucosa, mesenchymal stem cells, endothelial progenitor cells, embryonic stem cells, and induced pluripotent stem cells are available [45,46,47]. 

MSCs are a multipotent source of stem cells that can differentiate into cells from various lineages. MSCs from different sources such as adipose tissue, bone marrow, amniotic membrane, menstrual blood, urine, Wharton’s jelly, umbilical cord blood, etc., may be used [48,49]. The primary mechanism by which these cells may contribute to urethra defect repair is through paracrine secretions. It has been shown that the secretome of MSCs possesses immunomodulatory properties that can alleviate the hyperactive inflammation at the US site and reduce fibrotic reactions [50,51]. Furthermore, the proangiogenic function of these cells may facilitate the cell delivery vehicle’s vascularization, promote cell survival, and prevent graft failure [52,53]. Castiglione et al. showed that local injection of adipose-derived stem cells (ASCs) could successfully prevent US and urodynamic complications in a rat model of US [54]. Luo et al. reported that local injection of bone marrow MSCs, or their extracellular vesicles, alleviated US and prevented renal dysfunction in a rat model [55]. Although mal-differentiation of MSCs has not been reported in previous studies, none of these studies have investigated the adverse effects of urine exposure on mal-differentiation of MSCs. There is the possibility of MSCs carcinogenicity upon contact with urine, as these cells are not specialized to endure toxic substances in the urine [56]. 

Endothelial progenitor cells (EPCs) are circulating cells that attach to the site of ischemic injuries and participate in neovascularization. These cells share many surface markers with vascular endothelial cells and have been extensively studied to treat hypoxia/ischemia-induced tissue injuries [57]. Seeding these cells on the cell delivery vehicle may promote its vascularization and facilitate the tissue integration of the graft [58]. However, the paucity of these cells in the circulation poses a significant challenge for their clinical translation [59]. Chen et al. showed that co-transplantation of MSCs and EPCs via a decellularized human amniotic membrane could repair a 3-cm urethral defect in a canine model by preventing scar tissue formation and promoting angiogenesis [60].

The buccal mucosa is an epithelium lining the oral cavity from the inner surface of the cheeks and lips to their attachment with the alveolar ridge. The epithelial cells of this mucosal layer are stratified with high resistance against heat and physical damage [61,62]. The high healing activity of this epithelium may be due to the presence of progenitor cells in its basal layer. Cells isolated from the buccal mucosa are favored in urethroplasty procedures because of their ease of harvest and compatibility with wet environments [63,64]. However, complications such as donor site scarring, numbness, pain in opening the mouth, and mucosa dryness are this technique’s disadvantages [65]. Xie et al. isolated keratinocytes and fibroblast cells from the buccal mucosa and seeded them onto a silk fibroin matrix to develop a potential treatment modality for US. Cell-scaffold constructs showed no signs of US and formed a stratified epithelium in the injury site [66]. 

IPSCs are pluripotent stem cells generated by transfection of somatic cells with Myc, Oct3/4, Sox2, and Klf4 genes. The appeal toward these cells is rising, as these cells can differentiate into cells from three germ layers [67]. However, the differentiation potential of iPSCs depends on the origin of the cells, and it can be challenging to differentiate cells into the target lineage. On the other hand, these cells can potentially form teratoma and delivery of these cells by current cell delivery systems is challenging, because iPSCs require particular feeder layer substrates for adhesion and colonization [68,69]. On the positive side, urological cells can be produced via the differentiation of iPSCs in vitro. In this regard, Li et al. produced smooth muscle progenitor cells from iPSCs to repair urethra sphincter injury. The obtained cells expressed the phenotypical markers of smooth muscle cells and were engrafted at the injury site [70]. 

Embryonic stem cells are pluripotent cells isolated from the embryo’s inner cell mass. The isolation method of these cells poses a significant ethical challenge for their clinical translation. Furthermore, these cells may develop teratoma upon in vivo administration. Therefore, clinical use of these cells in medical practice seems unreachable in the coming years [71,72]. Like iPSCs, embryonic stem cells have potential applicability in urethra defects repair by differentiation into urothelial cells and smooth muscle cells. In this framework, smooth muscle cells were produced by differentiation of human embryonic stem cells and studied for their ability to restore urethra sphincter dysfunction following injury. This approach could significantly improve the sphincter function with no adverse effects [73]. Currently, preclinical data regarding the application of iPSCs and embryonic stem cells in the US treatment is lacking. Nevertheless, we believe that these cells have tremendous potential to treat US. However, various safety checks and long-term follow-up studies must be implemented before any clinical trial can occur. Furthermore, the ethical issues with the use of embryonic stem cells should be kept in mind.

The urothelium layer of the urethra is exposed to urine and protects the deeper compartments against urinary extravasation. Therefore, this layer is the first line of defense against the potential US. The success of any tissue-engineered urethra relies on perfectly mimicking this layer on the construct’s lumen. Otherwise, the US recurrence would be inevitable [74,75]. Three different factors guarantee the impermeability of this layer to urine. First, the asymmetric membrane structure of the umbrella cells consists of uroplakin plaque, which prevents direct urine penetration. Second, the occluding junctions between the upper cells block the paracellular path for urinary extravasation. Finally, the glycocalyx of these cells helps in the development of the urine–blood barrier [8].

The healing function of urothelial cells to treat urethral defects has been well-documented in previous studies [45,61,76]. Sievert et al. used a collagen type I carrier system to transplant urothelial cells into a Minipig model of urethral defect. Results showed that the transplanted cells successfully homed to the injury site without any rejection or inflammatory responses. Immunofluorescence studies showed that the transplanted cells had preserved their phenotypical markers and developed tight junctions with the resident cells [77]. Despite promising results, applying autologous sources of urothelial cells may be challenging. Low isolation yield, complex culture procedures, maintenance of urothelial progenitor cells stemness, and compromising the urothelium’s integrity upon cell harvesting are to be considered [75,78].

Cell-seeded scaffolds push the regeneration response of the injured urethra toward the natural healing response in small-sized injuries [14]. However, the lack of an organized muscular layer is the principal disadvantage of many recent studies. Urethra smooth muscle cells preserve the contractility of the channel and facilitate urine’s easy passage [79,80]. Therefore, a smooth muscle cell layer on the outer surface of the cell delivery system is crucial. In this context, Silva et al. compared the healing function of smooth muscle cells-seeded collagen tubes with cell-free ones. They observed that cell-delivering scaffolds had a significantly lower rate of stricture and infiltration of polymorphonuclear cells than their cell-free counterparts [81].

It seems that the use of urothelial cells on the luminal surface and a smooth muscle layer on the outer surface of the urethral grafts is the key to the success of a cell delivery system for US treatment. However, factors such as ethical issues, mal-differentiation of the seeded cells, availability of the cell source, and the effects of urine exposure on cell survival/differentiation need to be kept in mind. Furthermore, the use of non-specific cells may alter the tissue-engineered construct’s function in a long-term perspective. 

## 4. Bioactive Agents That Can Prevent US

Drug-delivering urethra grafts are gaining momentum to prevent US recurrence [82]. In this framework, various drugs such as, mitomycin C [83], inhibitors of the Wnt/β-catenin signaling pathway (ICG-001, IWR-1, and PRI-724) [84], TGF-β signaling pathway inhibitors (EW-7197) [85], triamcinolone [86], methylprednisolone [87], and captopril have been explored to prevent US (for more information, please see reference [88]).

Paclitaxel is a lipophilic chemotherapy medication that is used to treat various types of cancers. Recently, its anti-fibrotic potential has been utilized in urological procedures to prevent fibrotic reactions [89]. For instance, Zhang et al. showed that intraperitoneal injection of Paclitaxel could alleviate tubulointerstitial fibrosis via suppressing the TGF-β/Smad signaling pathway [90]. In another study, Zhang et al. showed that Paclitaxel could mitigate the activation of renal interstitial fibroblast cells by suppressing STAT3 signaling [91]. Recently, paclitaxel-eluting stents have been proposed to prevent US recurrence. Virasoro et al. followed-up on the efficacy and safety of a paclitaxel-coated Optilume™ balloon in 53 male subjects (Plymouth, MN, USA). Twelve-month follow-up showed that this system was safe and could significantly prevent US recurrence [92]. Although this strategy effectively prevented US recurrence, coating drugs on a balloon with or without hydrogel systems is not effective in controlling drug release. In this technique, the incorporated drug is usually released in a burst manner [93]. However, we need a sustained drug delivery system to prevent US in the long-term applications. 

Mitomycin C is a chemotherapeutic agent used to combat cancers such as breast, gastrointestinal, and bladder cancer. This drug is a potent DNA cross-linker and prevents DNA transcription into mRNA, reducing the cancer cell’s ability to synthesize proteins [94,95]. In the context of urethral defect repair, the anti-fibrotic function of mitomycin c has caught the attention of urologists. In this regard, Mazdak et al. investigated the anti-US potential of mitomycin C in a randomized clinical trial. Forty patients with the anterior US were included in this study, twenty of whom received urethral submucosal mitomycin C injection. Results showed that US recurrence rate in patients treated with mitomycin C was significantly lower than the control group [96]. 

Paclitaxel and mitomycin C have the merits of being used in clinical practice. However, given their anti-proliferative functions, incorporating these drugs into the cell delivery system may suppress cell proliferation and ECM secretion. Therefore, co-delivery of these drugs with the cell delivery platforms is not reasonable.

**Figure 2:**
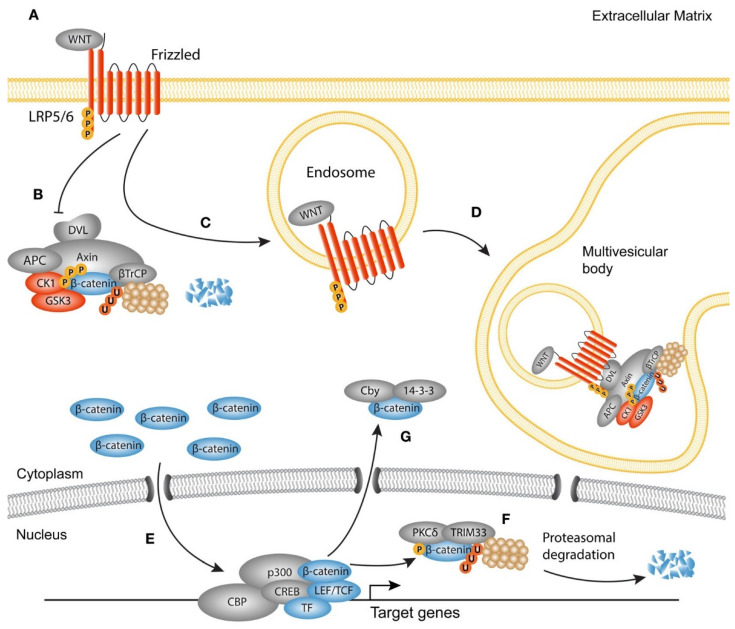
Schematic illustration representing the role of Wnt/β-catenin signaling pathway in fibrosis. (**A**) shows the binding of Wnt to the frizzled receptor that forms a complex with LRP. (**B**) shows the ubiquitination of the degeneration complex composed of DVL, Axin, APC, GSK3, and CK1. (**C**) shows the internalization of the receptor complex into the endosomal vesicles that causes the sequestration of the degeneration complex and suppression of GSK3. (**D**) GSK3 is then transferred to MVB and protects cytoplasmic β-catenin against proteasome degradation. In section (**E**), β-catenin is then carried to the nucleus and interacts with various transcription proteins such as LEF/TCF, p300, and CBP. (**F**) shows the termination of the signaling pathway. PKC-6 phosphorylates the β-catenin, making it a target for ubiquitination with (TRIM)33. Then, the ubiquitinated β-catenin is degraded in the proteasome complex. (**G**) Wnt/β-catenin signaling pathway may also end with 14-3-3ζ and Chibby (Cby). Abbreviations: Lipoprotein-related-receptor protein (LRP), dishevelled (DVL), adenomatous polyposis coli (APC), glycogen synthase kinase 3 (GSK3), and casein kinase 1(CK1), T cell factor/lymphoid enhancer factor family (LEF/TCF), protein kinase C (PKC)δ, tripartite motif (TRIM)33, CREB-binding protein (CBP). Adopted from reference [99].

The role of the Wnt/β-catenin signaling pathway in fibrosis has been shown in previous studies [97]. Soluble Wnt proteins bind to their cell surface receptors, known as Frizzled, and trigger intracellular signaling pathways, leading to the regulation of various target genes expression involved in the fibrotic reactions (Figure 2) [98].

Indeed, disorders in the Wnt/β-catenin signaling pathway have turned out to be correlated with fibrogenesis in different tissues. Therefore, this signaling system may be a prime target for anti-fibrotic drugs [100,101]. Inhibitors of the Wnt/β-catenin signaling pathway, including ICG-001, IWR-1, and PRI-724, bind to different components of this signaling pathway and mitigate fibrosis. For instance, ICG-001 binds to CBP and acts as an antagonist for the Wnt/β-catenin signaling pathway. Choi et al. investigated the anti-fibrosis potency of ICG-001, IWR-1, and PRI-724 in a rat model of US. They showed that rats treated with ICG-001 or PRI-724 demonstrated a significantly lower level of US and tissue expression levels of collagen type I and alpha-smooth muscle actin genes [84]. In addition to the Wnt/β-catenin signaling pathway, other signaling systems such as YAP/TAZ and TGF-β signaling are involved in organ fibrosis. Therefore, antagonist drugs against these signaling pathways may also be of therapeutic value in treating US (for more information, please see reference [99]). For example, the TGF-β signaling pathway inhibitor, EW-7197, has been found to prevent fibrosis in different disease models [102,103].

Triamcinolone is a glucocorticoid, utilized in the clinic to suppress hyperactivity of inflammatory responses. This drug prevents US by decreasing ECM component synthesis and suppressing pro-fibrosis inflammatory mediators [104]. In a randomized clinical trial, Mazdak et al. showed that submucosal injection of triamcinolone (40 mg) could significantly reduce US recurrence after 12 months of internal urethrotomy [105]. Zhou et al. showed that co-administration of triamcinolone and 5-fluorouracil significantly reduced US in a rat model by upregulating miR-192-5p expression [106]. Methylprednisolone is another glucocorticoid that has found application in the management of US. Abdallah et al. showed that intra-urethral injection of methylprednisolone could significantly prevent US recurrence following DVIU [87]. 

Studies on US glucocorticoid treatment have focused on local injection following urethrotomy. However, this administration method does not provide long-term drug bioavailability. On the other hand, drug delivery by polymeric urethral grafts provides better control over drug release profile [107]. It should be noted that the incorporation of immunosuppressive drugs into the matrix of urethral grafts may increase the risk of urinary tract infection (UTI) or the development of malignancies [108]. However, preclinical or clinical data to support this theory are lacking. 

Angiotensin II promotes tissue fibrosis via triggering the fibrotic reactions [109]. Captopril can inhibit US via blocking angiotensin-converting enzyme and production of Angiotensin II. In this context, Kumiawan et al. investigated the anti-fibrotic function of captopril-loaded gel in a rabbit model of US. They showed that transurethral injection of captopril gel significantly reduced US by decreasing the tissue expression levels of TGF-β1 and connective tissue growth factor (CTGF) [110]. In a phase II clinical trial, Shirazi et al. showed that captopril gel could prevent US recurrence and increased urine flow without adverse reactions [111].

Generally, these drugs can be loaded into the electrospun scaffolds to combat US recurrence. However, the effects of the electrospinning process on the loaded drugs’ biological activity should be considered [18]. 

## 5. General Characteristics of Cell and Drug Delivery Systems to Treat Urethra Defects

The natural healing process in the urethra involves the interplay between multiple role players, such as growth factors, cytokines, cell-to-cell contacts, secretome of resident and migratory cells, and various signaling pathways [112,113,114]. Therefore, polymer-only scaffolds are unsuitable for repairing urethral defects and must be engineered to provide multiple biological and biophysical cues [53]. In this framework, significant strides have been made to incorporate different cells and bioactive agents into the matrix of tissue-engineered scaffolds to improve their healing activity (Table 1).

Although cells can be directly injected at the site of injury, various complications such as cell migration, lymphatic drainage, low cell survival, and off-target homing compromise the healing function of the administered cells [115]. In contrast, delivery via a carrier system can protect cells against environmental factors, localize them at the site of injury, and improve their biological activity [116]. However, the complexity of the native cells niche requires a versatile approach to delicately engineer a delivery system that provides essential cues for cells’ survival, colonization, and differentiation [117,118]. In particular, stem cell delivery via biomaterials-based delivery systems is challenging because the differentiation program of stem cells is governed by various parameters such as the biophysical properties of their microenvironment, spatiotemporal exposure to various biological cues, and their surface receptors’ interactions with biological macromolecules [119]. In the context of urethral tissue engineering, different cell delivery systems such as hydrogels, porous scaffolds, decellularized tissues, fibrous scaffolds, bioprinted scaffolds, and self-assembled ECM are available [120,121]. 

An ideal cell delivery vehicle for urethra tissue repair should possess various functionalities such as sufficient mechanical strength to shield cells against external forces, suturability, ability to preserve cells’ biological function through providing biochemical/biophysical cues, biocompatibility, appropriate biodegradation time in a way that its degradation keeps pace with the tissue regeneration process, selective permeability to urine and nutrients, and a porous structure to allow cell ingrowth and vascularization [10,53,61,78]. However, manufacturing such a complex system with the existing technology looks pretty demanding. 

Generally, natural polymers-based cell delivery systems have excellent biocompatibility, but their poor biodegradation rate and low mechanical strength hamper their cell delivery applications in urethra defects repair [122]. On the other hand, synthetic polymers have excellent mechanical properties and tunable biodegradation. However, due to the lack of cell adhesion moieties and high hydrophobicity, cell tendency toward these polymers is poor, resulting in a significant reduction in cell survival [123,124]. Therefore, one can fabricate a hybrid delivery system by blending synthetic and natural polymers. In this way, the delivery system supports cells’ survival and colonization; while buying enough time for the resident cells to replace the artificial scaffold with their ECM [125].

**Figure 3:**
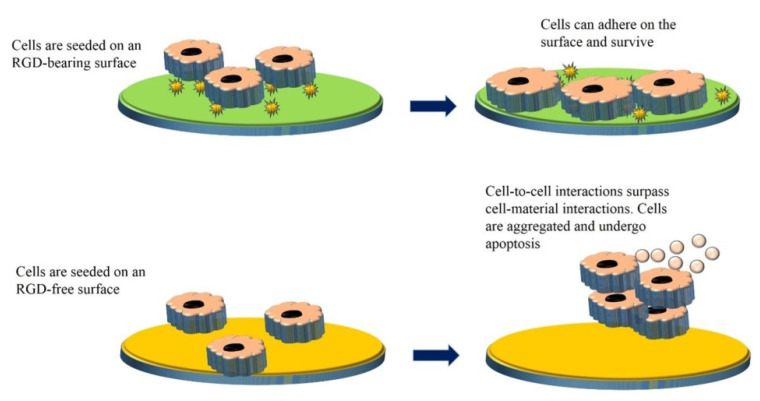
Schematic illustration representing the interaction of cells with RGD-free and RGD-incorporated surfaces.

The delivery system for adherence-dependent cells should provide cell recognition sites for cell adhesion and survival; otherwise, the cell-to-cell interactions will surpass cell–material interactions that cause the cells aggregation within the delivery system and their apoptosis (Figure 3) [119]. Integrins are principle cell surface receptors that mediate cell binding to ECM components such as collagen, fibronectin, and vitronectin. These polymers possess RGD moieties in their structure, and integrins bind to these peptides. Therefore, delivery systems for these cells should contain RGD-bearing polymers or be functionalized with RGD moieties [126].

Selective permeability to urine and nutrients is the crucial factor in the success of a cell delivery vehicle in urethral defects repair. While the diffusion of nutrients is essential for the survival of the seeded cells, the delivery system should prevent urinary extravasation and subsequent fibrotic reaction [127]. However, current scaffold fabrication methods fail to produce such a smart membrane. Therefore, the tissue–urine barrier can only be achieved via effective differentiation of urothelial cells in the cell-delivery system [128].

An appropriate biodegradation rate guarantees that the delivery system will preserve its structural integrity until the cells can build their niche. A high biodegradation rate may cause the graft’s failure and swelling. On the other hand, a slow biodegradation rate may cause foreign body reactions and chronic pain after graft’s implantation [129].

Incorporating bioactive agents can enhance the healing activity of polymer-only scaffolds. In this context, various growth factors, signaling molecules, cytokines, and small drug molecules are available to enhance the bioactivity of the urethral grafts [130,131]. However, the spatiotemporal control over the release of these agents is of prime importance. This issue is fundamental when fabricating a dual-function delivery system for cells and bioactive agents. In this scenario, the dose-dependent effects of the bioactive agents on cellular behavior must be investigated [93]. An ideal drug carrier for urethra tissue repair should encapsulate the bioactive agents without damaging their biological activity, possess a sustained drug release profile, preserve its structural integrity in long-term applications, possess a homogenous drug distribution all over the matrix, and have a tunable drug release [132,133,134]. 

**Figure 4:**
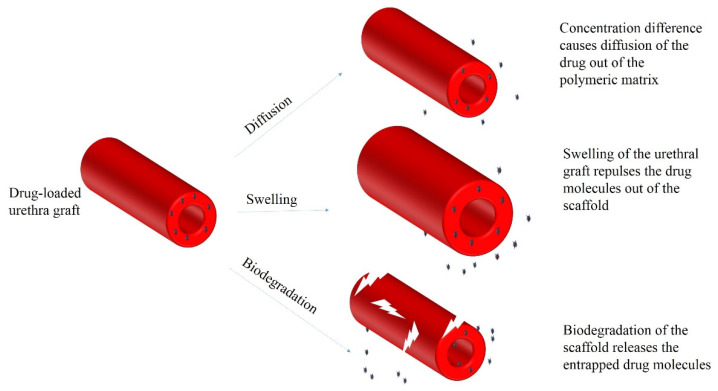
Schematic illustration representing the mechanisms involved in drug release from polymeric urethra grafts.

Different mechanisms govern the release profile of bioactive agents from polymeric scaffolds. First, the diffusion of the bioactive agents due to the concentration difference is the primary driving force in the release profile. Second, the swelling of the polymeric matrix upon contact with the physiological fluids may repulse the bioactive agents out of the matrix. Finally, gradual degradation of the polymeric matrix may explain the sustained drug release from the carrier system (Figure 4) [135]. 

Table 1 summarizes the recent applications of different drug and cell delivery systems to treat urethral defects. As shown, most of the studies have been focused on hydrogel systems or decellularized scaffolds. Poor mechanical strength and fast biodegradation are the significant disadvantages of hydrogel systems for cell or drug delivery applications. Although these drawbacks can be addressed by increasing the cross-linking degree, excessive cross-linking may sacrifice the hydrogel’s injectability [136,137]. On the other hand, decellularized scaffolds cannot be efficiently functionalized with anti-fibrotic reagents. Therefore, the ideal cell or drug delivery system for urethral defects repair should be easily sutured at the injury site, have functionalization capability, and allow urine passage [14,46]. Fortunately, electrospun scaffolds meet all these criteria.

**Table 1:** Summary of previous applications of cell and drug delivery systems to treat urethra defects.

Polymer/s.	Delivery System	Cell/Drug Type	Fabrication Method	Cell Type/s for In Vitro Study	In Vivo Model	Experimental Results	References
Polycaprolactone and fibrin	Hydrogel	Urothelial cells and smooth muscle cells	3D printing	Urothelial cells and smooth muscle cells	-	The produced delivery system had comparable mechanical strength with rabbit urethra and supported cell viability up to 7 days after printing	[138]
Propylene glycol	Hydrogel	Mitomycin C	Cross-linking	-	Clinical trial	Mitomycin C-loaded hydrogel could significantly reduce the recurrence of US after internal urethrotomy	[139]
Gelatin methacrylate and pure collagen	Bioprinted scaffold	Bladder smooth muscle cells	Bioprinting	Bladder smooth muscle cells	-	Cells stayed viable in the printed scaffolds, and cell density increased over time	[140]
Poly l lactic acid, poly D,L-lactic-co-glycolic acid, and poly(L-lactic acid-co-ε-caprolactone)	Porous sponge	Adipose-derived stem cells	Lyophilization	Adipose-derived stem cells	New Zealand white rabbit model of urethra defect	Hypoxia-preconditioned stem cells delivered via the porous scaffolds could successfully repair urethral defects and induced a robust angiogenesis	[141]
Poly [N-isopropyl acry-lamide-co-n-butyl methacrylate] poly [NIPAAm-co-BMA]) and hydrophilic blocks (polyethylene glycol)	Hydrogel	Buccal epithelial cells	Sol-gel transition was obtained by changing the temperature.	Buccal epithelial cells	Clinical trial	The treated patients void well with a normal mean peak flow rate. Two of the six patients showed recurrent stricture at 18 and 24 months after treatment	[142]
TGP	Hydrogel	Buccal mucosal epithelial cells	Thermo-reversible gelation	Buccal mucosal epithelial cells	Japanese white male rabbit model of urethra defect	Cells stayed viable in the hydrogel system and differentiated into fibroblast-like cells. Cell-loaded hydrogel system repaired urethra defects and cells engrafted at the injury site	[143]
No materials were used	tubular scaffold	Human mesenchymal stem cells	Self-assembly	Human mesenchymal stem cells	Nude rat model	scaffolds showed two layers of cells and no stricture after implantation into the nude rat	[144]
Natural ECM	Decellularized bladder matrices obtained from lamina propria	Autologous bladder epithelial and smooth muscle cells	Decellularization	Autologous bladder epithelial and smooth muscle cells	Rabbit model of anterior penile urethra defect	Cell-seeded tubular matrices showed a wide urethral caliber with no strictures. In addition, a transitional cell layer was formed in the cell-seeded matrix group, and the newly developed urethra showed contractility	[145]
Silk fibroin and a nanoporous bacterial cellulose	Porous Bilayer scaffold	Lingual Keratinocytes and muscle cells	Freeze-drying and self-assembling	Lingual keratinocytes and muscle cells	Canine model of urethra defect	Microstructure studies showed that the seeded cells could adhere to the scaffolds. Cell-seeded urethral grafts showed superior healing function over cell-free ones	[146]
Bacterial cellulose	3D porous scaffold	Lingual keratinocytes	Biosynthesis via bacterial species	Lingual keratinocytes	New Zealand White male rabbit model of urethra defect	In scaffolds seeded with lingual keratinocytes, the caliber of the urethras was wide open and a continuous epithelium was formed	[147]
Natural ECM	Decellularized human amniotic scaffolds	Allogeneic bone marrow mesenchymal cells and/or endothelial progenitor cells	Decellularization	Bone marrow mesenchymal cells and/or endothelial progenitor cells	Canine model of circumferential urethral defect	Animals treated with cell-seeded scaffolds showed unhindered urination and wide open urethra caliber. Furthermore, extensive vascularization was observed in this group	[60]
Natural ECM	3-D porous small intestinal submucosa	Urothelial and smooth muscle cells that were produced from the differentiation of urine-derived stem cells.	Decellularization	Urine-derived stem cells differentiated into urothelial cells and smooth muscle cells.	Cell-seeded scaffolds were implanted into Athymic mice	The seeded cells developed uniform layers on the scaffold and penetrated deep into the inner parts	[148]
Poly-D,L-lactide-co-εcaprolactone	Bilayer polymeric matrix	Allogenic mesenchymal stem cells	Casting and air drying	Mesenchymal stem cells	Chinchilla rabbit model of urethra defect	Cell-seeded scaffolds showed integration with the urethra tissue with no adverse tissue reactions. Delivered cells expressed cytokeratin marker AE1/AE3, implying their potential differentiation into neo-urothelium	[149]
Natural ECM	Acellular matrix	Endothelial progenitor cells that secrete antibiotic peptide LL37	Decellularization	Endothelial progenitor cells	New Zealand white Male rabbit model of urethra defect	Antipoetic-delivering cells seeded on the acellular matrix could successfully repair critical-sized urethra defects	[150]
Gelatin, poly l lactic acid, and silk fibroin	Porous tubular scaffolds	Mitomycin C and epidermal growth factor	Freeze drying	Urethral epithelial cells and urethral scar-derived fibroblast cells	-	The proportion of Urethral epithelial cells was significantly increased when cultured on the drug-loaded system	[151]
Poly-l-lactic acid and poly-dl-lactic acid	Tubular scaffold	Paclitaxel	Casting	-	Male rabbit model of urethra defect	The drug-eluting stent could successfully prevent US, reduced inflammation, and alleviated fibrotic reactions	[152]
Poly-L189 lactide-co-caprolactone (PLC) and Polyethylene glycol diacrylate (PEGDA)	Polyurethane double pig-tailed ureteric stent spray-coated with Mitomycin C-loaded PLC and overlaid with PEGDA hydrogel.	Mitomycin C	Spray coating and cross-linking	HBdSF cells	Porcine model	The developed system released the loaded drug sustainably and could deliver the drug to urothelium with no adverse effects	[153]
Collagen	A synthetic catheter coated with collagen	Insulin-like growth factor 1 (IGF-1)	Coating on a synthetic catheter	HUEpCs cell line	Japanese white rabbit model of urethra defect	Animals treated with IGF-1/collagen-impregnated catheters had significantly bigger urethra caliber than other groups	[154]

## 6. Principles of Electrospinning

**Figure 5:**
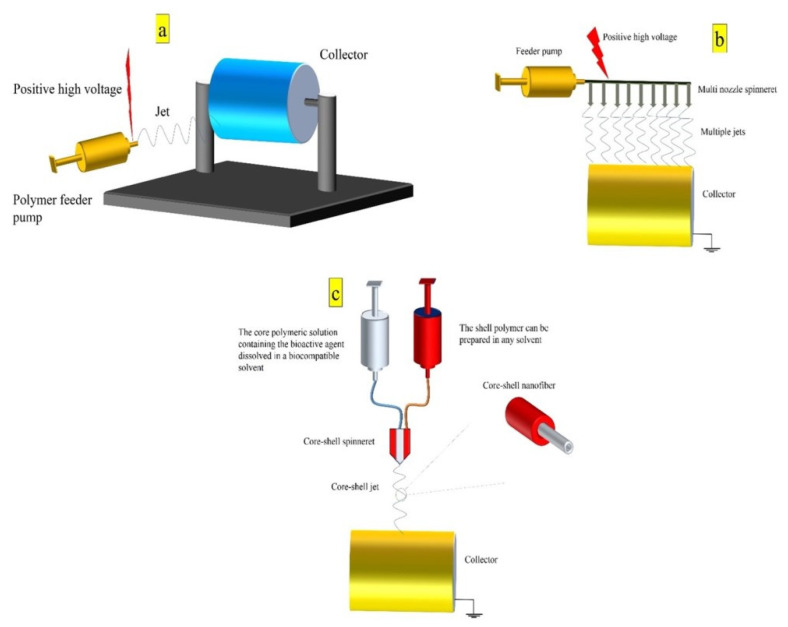
Schematic illustration representing (**a**) the conventional electrospinning device, (**b**) the multi-nozzle electrospinning method to increase the production yield, and (**c**) a core-shell electrospinning method to preserve the biological activity of bioactive agents.

The electrospinning technology is based on spinning a conductive droplet of a polymeric solution under a high voltage in an electrostatic field. In this technology, polymers are dissolved in a solvent that are then loaded into a syringe pump with a metal needle [155,156]. A positive high voltage is then applied to the needle and the solution is fed under a constant rate (usually 0.5–2 mL/h) (Figure 5a) [157]. The solvent gets charged under the electrostatic field when exposed to a positive high voltage. By increasing the magnitude of high voltage, the electrostatic forces gain sufficient energy to surpass surface tension charges and the polymeric solution turns into a conical shape, known as the Taylor cone [15,17]. Then, the Taylor cone tends to eject toward the collecting mandrel and starts to form a polymeric jet. The solvent in the polymeric jet vaporizes and increases its surface charge. Then, the jet destabilizes and separates into multiple jets while travelling toward the collector [16,158].

The conventional electrospinning method has a low production yield and cannot be utilized for the mass production of fibrous scaffolds. This drawback can be addressed by increasing the number of spinnerets (Figure 5b) [159]. Furthermore, many solvent systems are incompatible with bioactive agents and damage their structure and function. Notably, growth factors are susceptible to organic solvents and lose their biological activity upon contact with these chemicals [18]. Core-shell electrospinning method can address this issue. In this method, the bioactive agent is dissolved in a compatible solvent (usually aqueous solvents) and spun in the core. At the same time, the shell part of the fibers can be a polymeric solution with any solvent system (Figure 5c) [160].

Various properties of electrospun fibers, such as their alignment, surface topography, morphology, average diameter, and porosity can be tuned by altering the fabrication parameters. In this regard, the effects of different factors on these properties have been studied (Table 2).

## 7. Principles of Cell Delivery via Electrospun Scaffolds

**Figure 6:**
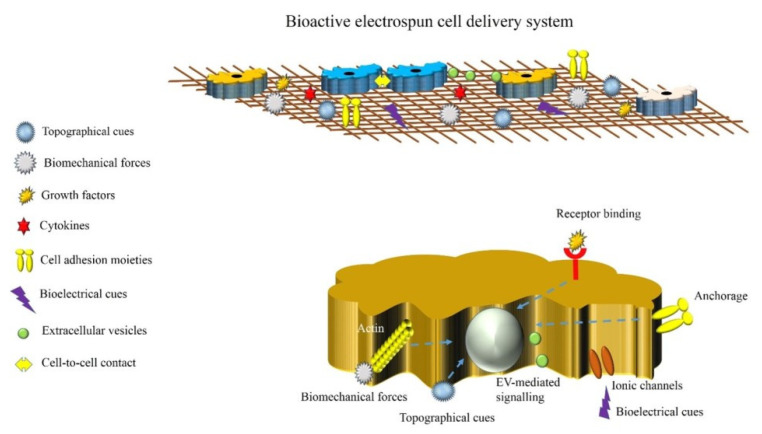
Schematic illustration representing the components of a bioactive electrospun cell delivery system. Cells bind to different niche components with surface receptors, thus activating the cell anchorage-dependent signaling pathways. Cells respond to topographical cues and biomechanical forces by altering their cytoskeleton and various signaling pathways. EVs derived from neighboring or distant cells are internalized into the seeded cells and mediate intercellular communications. Bioelectrical cues regulate cellular function by ionic flows from ionic channels. Growth factors and cytokines bind to cellular receptors and convey messages to the cytoplasm or nucleus by a diverse range of signaling pathways.

The natural niche of cells is composed of tissue-specific microenvironmental factors that control cells’ behavior and preserve their biological functions. In this context, a diverse range of biochemical and biophysical cues such as growth factors, cytokines, the architecture of ECM, the composition of ECM, mechanical properties, bioelectrical cues, surface topography, cell-to-cell contacts, and intercellular communications by extracellular vesicles (EVs) have been found to affect cellular behavior and function [173,174,175]. Therefore, an ideal electrospun cell delivery vehicle should mimic this complex network (Figure 6).

Growth factors and cytokines are bound to ECM components, and their exposure to cells is tightly regulated. In particular, fibroblast growth factor family members adhere to ECM’s heparan sulphate and are released on demand [176,177]. Furthermore, the ECM can serve as a reservoir for different ligands, developmental factors, and bioactive fragments [178]. Although electrospun fibers have a high surface-to-volume ratio and can be functionalized with these biochemical cues, the complexity of interactions between these factors and ECM components poses a significant challenge in developing an artificial niche by electrospinning method. 

The web-like structure of electrospun scaffolds is highly similar to ECM architecture. However, the composition of natural ECM cannot be recapitulated by electrospinning [158,179]. The natural ECM comprises various components such as collagen, elastin, proteoglycans, and glycoproteins. While a small proportion of these components can be electrospun by blending with other polymers, electrospinning of only these components is not possible [53]. Therefore, mimicking the exact composition of natural ECM by the electrospinning method cannot be achieved. ECM is not just the sum of molecules such as collagen, elastin, or GAG, but an organized system with a fine network controlled by several dozen other proteins to ensure its function. For example, elastin molecules are entirely different from the elastic network and the elastic function, and we can have much elastin without having any elastic network [180]. 

Cells perceive the mechanical properties of their microenvironment by various surface receptors, focal adhesions, nuclear signaling factors, and mechano-sensors [181,182]. Given the importance of the mechanical cues in controlling cellular behavior, the mechanical properties mismatch between native tissues ECM and electrospun scaffolds need to be addressed. This difference in mechanical properties can be explained by composition discrepancies, hydration status, and degree of cross-linking [53]. Furthermore, the mechanical properties of electrospun scaffolds can be tuned by changing the polymer type, altering the degree of cross-linking, and incorporating filler materials [183]. 

Bioelectrical cues convey regulatory messages to the nucleus by endogenous ionic flows. These signaling systems affect cellular behavior, regulate tissue regeneration mechanisms, and are involved in the initial development of organs [184]. Unfortunately, many of the existing polymers for urethra tissue engineering are not electrically conductive. This property can be imparted to electrospun cell delivery systems by incorporating conductive filler materials such as carbon nanotubes or metallic nanoparticles [185]. However, the cell delivery system for urethra tissue repair does need to be conductive. 

Seeded cells on electrospun cell delivery systems may respond to their surrounding surface topography by expressing different focal adhesion complexes. For example, cells rearrange their cytoskeletal actin in response to changes in surface topography. In this regard, the essential role of Rho-associated kinases has been proven in previous studies [186,187,188]. Furthermore, focal adhesion kinases (FAK) mediate cellular responses to surface topographies [189]. However, identifying tissue-specific surface topographies and their introduction onto the electrospun cell delivery systems are challenging. Generally, electrospun scaffolds have a smooth surface and cannot mimic this feature of cell niche. Therefore, new fabrication methods need to be developed to address this issue. 

Various surface proteins on the neighboring cells mediate the intercellular communications. Furthermore, cells send messages to their neighboring cells by EVs. These membrane-packed vesicles contain miRNAs, mRNA, DNA, and proteins that can cause a behavioral change in the recipient cells [190,191]. The composition and function of these vesicles depend on their microenvironment. For instance, it has been shown that the cells exposed to hypoxic conditions secrete EVs with a higher content of proangiogenic miRNAs [192,193]. Therefore, the electrospun cell delivery system may potentially affect this signaling system. However, no previous study has investigated this phenomenon so far. 

## 8. Principles of Drug Delivery with Electrospun Scaffolds

**Figure 7:**
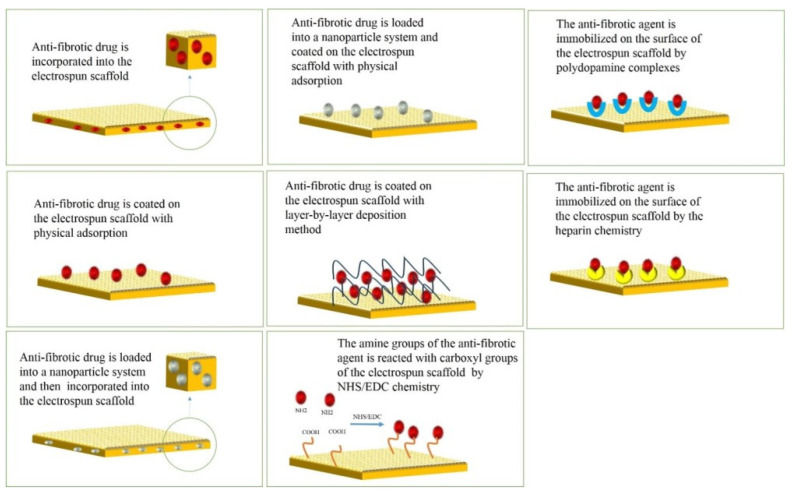
Schematic illustration representing the functionalization of electrospun scaffolds with various drug loading technologies.

The complexity of US pathophysiology requires a versatile approach to develop effective treatment strategies. Even though electrospun scaffolds recapitulate some features of the native urethra tissue, their bioactivity is not sufficient to prevent US recurrence [16,194]. Therefore, anti-fibrotic bioactive agents should be incorporated into their matrix or immobilized onto their surface. In this regard, various methods such as blending with the polymeric solution, physical adsorption, use of nanoparticles, layer-by-layer surface coating, and surface immobilization technologies have been proposed to functionalize the electrospun scaffolds (Figure 7) [93,195].

In the physical encapsulation method, the anti-fibrotic drug is blended with the polymeric solution, and the resulting mixture is finally electrospun. Although this method is straightforward, optimizing drug/polymer solution in higher drug concentrations is challenging. Furthermore, the solvents may damage the structure and biological activity of the loaded drug [18,196,197].

The physical adsorption of anti-fibrotic drugs on the surface of electrospun scaffolds is mediated by electrostatic interactions and van der Waals forces. Therefore, this method can preserve the biological function of the drug. However, burst drug release is the main disadvantage of this method in drug loading [195].

Nanoparticle systems are advanced drug carriers with a high surface-to-volume ratio, high encapsulation yield, and suntanned drug release profile [198]. These drug carriers can be utilized to functionalize the electrospun scaffolds. For example, the anti-fibrotic drug can be encapsulated into the nanoparticle system and then electrospun with the polymeric solution [199]. An alternative strategy would be the physical adsorption of the drug-loaded nanoparticle carrier on the electrospun platform [155].

In the layer-by-layer deposition method, the anti-fibrotic drug is entrapped into multilayer polymeric films, and these layers are deposited onto the electrospun carrier [200]. Various polymers such as proteins, polyelectrolytes, and micelles may be used. In this system, various forces such as electrostatic, hydrogen bonding, hydrophobic, and chemical bonds may stabilize the surface coating [201]. By altering the thickness of the multilayer film or polymer type, tunable release kinetic of the anti-fibrotic drug can be obtained [202,203].

The chemical immobilization of the anti-fibrotic drug on the electrospun scaffold provides better control over the drug release profile. In this method, a cross-linking reagent such as NHS/EDC mediates the covalent bonding between the functional groups on the electrospun scaffold’s surface and the functional groups of the drug molecule [204,205]. 

The inherent adhesive capability of mussels has inspired a new surface functionalization technology. This adhesion potential can be attributed to the presence of 3,4-dihydroxy-L-phenylalanine (DOPA) and lysine amino acids in the structure of mussels. Furthermore, polydopamine complexes containing catechol and amine groups have the same adhesion activities [206]. Therefore, surface coating of anti-fibrotic drugs on electrospun scaffolds can be performed using polydopamine structures [207].

In natural ECM, heparan sulfate binds to various growth factors, signaling molecules, and bioactive peptides. This binding capacity can mediate the surface immobilization of anti-fibrotic agents on the electrospun scaffolds [208].

The diversity of these drug loading technologies mirrors the considerable potential of electrospun scaffolds for drug delivery applications. However, in the context of anti-fibrotic drug delivery, most studies have focused on the physical encapsulation method.

## 9. Previous Applications of Electrospun Cell Delivery Systems to Treat Urethra Defects

The combination of electrospinning method with other scaffold fabrication technologies is a valuable strategy to produce constructs with higher similarity to urethra tissue. In this regard, electrospun polyurethane urea (PUU) fibrous membranes were coated with collagen hydrogel to produce a fibrous hydrogel (cPUU) [209]. This system could recapitulate the structure of urethra’s natural ECM, which is a fibrous construct embedded into a hydrogel ground substance. Furthermore, this system could successfully support the adhesion and growth of bladder smooth muscle cells. The healing activity of bladder smooth muscle cells delivered by this system was investigated in a rabbit model of urethral defect. In vivo evaluations showed that rabbits treated with cell-seeded scaffolds had significantly better urethra reconstruction than other experimental groups. In addition, complications such as stone formation, US recurrence, and fistula were significantly lower in cPUU scaffolds compared with control and PUU-only groups (For the figures, please see reference). Despite promising results, this delivery system did not meet the criteria of a bioactive cell delivery system. For instance, many essential biochemical and biophysical cues were not incorporated into this delivery vehicle.

Vascularization of tissue-engineered grafts is essential for their success in tissue regeneration. However, the small pore size of electrospun cell delivery systems impedes the ingrowth of small capillaries [210]. Niu et al. developed a novel approach to address this challenge. Amphiphilic PUU/polycaprolacton (PCL) nanofibrous scaffolds with a rapid vascularization function were fabricated [211]. Smooth muscle cells were seeded onto the outer and middle layers, while epithelial cells were seeded onto the inner surface. The results showed that the seeded cells grew and spread well on the scaffolds. While PUU-based scaffolds were conducive to expressing phenotypical markers of smooth muscle cells and epithelial cells, PCL-only scaffolds failed to provide such function. The developed system was implanted into a Beagle puppy model with an urethra defect. Functional analysis and histopathological examinations showed that cell-seeded PUU scaffolds were rich in ECM and cellular components, induced angiogenesis, promoted the lumen re-epithelialization, and had comparable functional recovery outcomes with the autograft group. In comparison, the PCL group had a negligible healing function. Biochemical and biophysical cues in the amphiphilic PUU scaffolds may explain their higher regenerative function compared with PCL-only scaffolds. In another study, Niu et al. used electrospun block polyurethane (PU-*alt*) scaffolds for co-delivery of urethral epithelial and smooth muscle cells [212]. The produced yarns supported the seeded cells’ adhesion, proliferation, and specific markers expression. The healing function of the autologous tissue-engineered urethra was investigated in a rabbit model of urethra defect. Results showed that scaffolds promoted the neovascularization process, induced oriented growth of smooth muscle cells, and improved the formation of the epithelial layer. Although these studies showed the controlled neovascularization by PUU scaffolds, incorporating the proangiogenic growth factors such as vascular endothelial growth factor (VEGF), basic fibroblast growth factor (b-FGF), platelet-derived growth factor (PDGF), etc., may provide better graft vascularization [213,214]. Furthermore, seeding the scaffolds with EPCs or MSCs may further improve the vascularization on the electrospun scaffolds [215].

**Figure 8:**
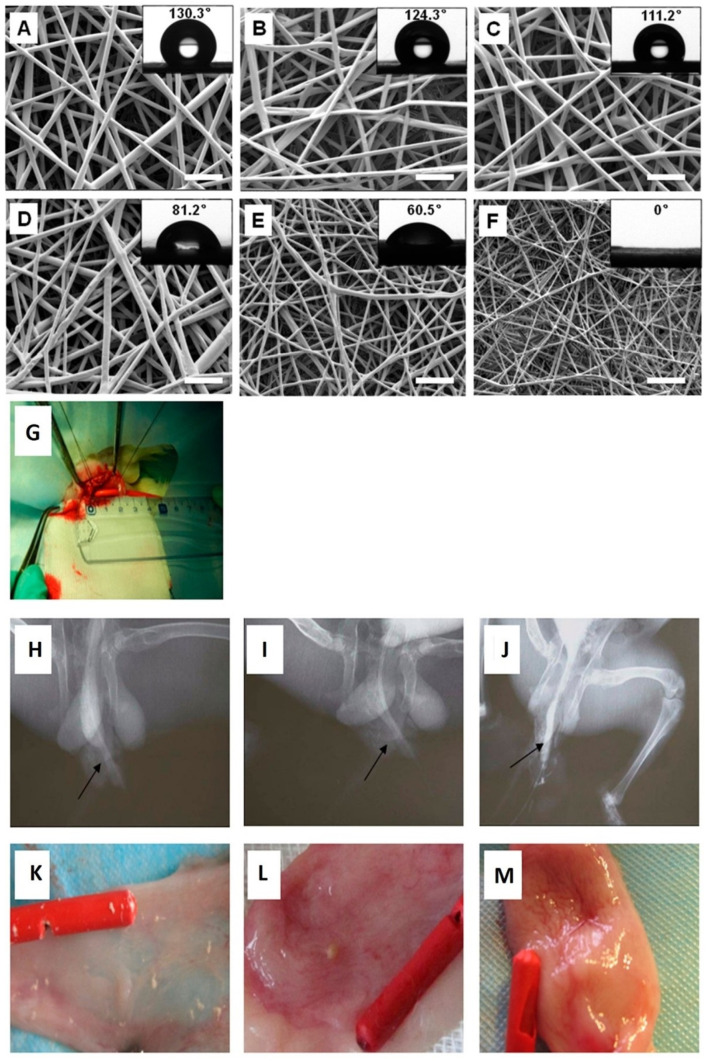
(**A**–**F**) shows SEM images of PLLA scaffolds with different PEG weight ratios: (**A**) 0%, (**B**) 10%, (**C**) 20%, (**D**) 30%, (**E**) 40%, (**F**) 50%. (**G**–**M**) show the implantation of PLLA/PEG/ hAMSCs in the rabbit model, and the repair of the urethra defect. (**G**): implantation process. (**H**–**J**): retrograde urethrograms of animals treated with the mock operation, PLLA/PEG scaffolds, and PLLA/PEG/hAMSCs, respectively. (**K**–**M**) show the urethra mucosa defect repair and calculi formation in rabbits treated with the mock operation, PLLA/PEG scaffolds, and PLLA/PEG/hAMSCs, respectively. Adopted from reference [219]. Scale bar 10 μm.

**Figure 9:**
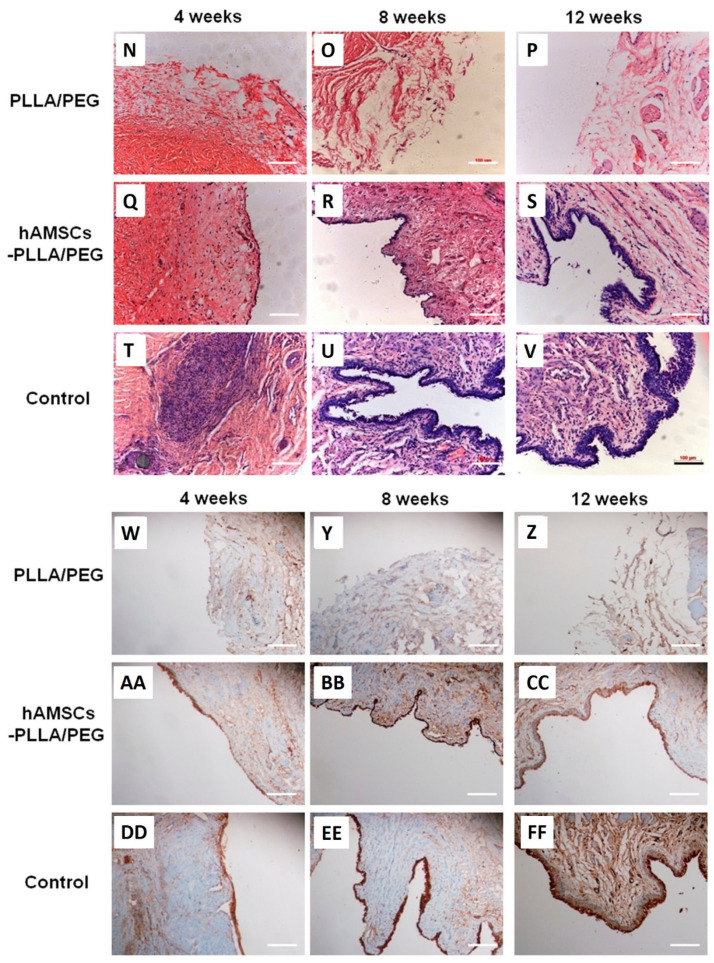
(**N**–**V**) show the H & E staining images of animals treated with different scaffolds at weeks 4, 8, and 12. (**W**–**FF**) show the IHC staining images of urethra tissues against the AE1/AE3 marker at weeks 4, 8, and 12. Adopted from reference [219]. Scale bars, 100 μm.

One of the main challenges of cell-based therapies is isolating autologous cells with minimally invasive methods. Adipose-derived MSCs (or ASCs) are an abundant and accessible source of stem cells that can be isolated from fat tissues with minimal donor site morbidity [216]. Wang et al. used electrospun poly lactic acid (PLA) scaffolds to transplant ASCs into a rabbit model of urethra defect [217]. The study showed that urethral stenosis was significantly lower in rabbits treated with ASC-seeded PLA membranes. Furthermore, histopathological examinations showed that this group had restored urethra structure with typical urothelial and muscular layers. Lack of cell recognition sites, high hydrophobicity, and acidic biodegradation residues are the main disadvantages of PLA-based cell delivery platforms [218]. This system can be improved by blending PLA with ECM-derived or hydrophilic polymers. These polymers possess cell recognition sites in their structure and are hydrophilic; therefore, they can promote cell adhesion and modulate the surface wettability of PLA-based scaffolds [53]. In this context, a hybrid cell delivery platform was produced by blending poly(ethylene glycol) (PEG) with PLLA [219]. The produced yarns had a fibrous structure. Furthermore, the water contact angle (an indicator of surface wettability) of PLLA scaffolds was decreased by increasing the PEG fraction (Figure 8A–F). PLLA/PEG scaffolds were seeded with human amniotic MSCs (hAMSCs) before implantation into a rabbit model of urethra defect. The study showed that hAMSCs could adhere and proliferate on the scaffolds. In vivo study showed that the animals treated with PLLA/PEG/hAMSCs showed no fistula or US in the urethrography studies or morphological observations (Figure 8G–M). Histopathological examinations showed that a multilayered urothelium was formed on the scaffolds for the PLLA/PEG/hAMSCs group. However, the urethral mucosa was still discontinuous (Figure 9N–V). Immunohistochemical studies showed that the defects treated with PLLA/PEG/hAMSCs were stained positive for AE1/AE3 marker, indicating that epithelial cells have been formed in this group. In comparison, other groups were stained negative for this marker (Figure 9W–FF). The seeded cells on the nanofibrous membranes may have promoted the urethral defect repair by releasing pro-healing growth factors, mitigating fibrosis via modulation of inflammatory responses, or increasing the graft’s vascularization.

EVs derived from stem cells can recapitulate the same regenerative responses observed in whole cell-based therapies. Exosomes are a subclass of EVs that are sized between 30 and 120 nm [220]. Wang et al. blended poly (L-lactide-co-caprolactone) (P(LLA-CL)) with collagen to produce a delivery platform for ASC-derived exosomes [221]. The developed system was not toxic against fibroblast cells and reduced the secretion of pro-inflammatory factors such as IL-6. In vivo study showed that the exosome-nanofibrous scaffold system prevented US and induced a multilayer epithelial tissue formation. Although EVs are an integral part of cells niches and their healing potential has been shown in various disease models [222], seeding cell delivery systems with functioning cells may provide a better healing response, because these cells can interact with their microenvironment and release EVs specific to the disease condition [193,223]. Furthermore, whole cells can remodel the tissue-engineered constructs and aid in their replacement with natural ECM [224]. 

Silk fibroin is a naturally occurring protein with numerous positive features for tissue engineering, such as excellent tensile strength, biodegradability, and nontoxicity [225]. Xie et al. used electrospun silk fibroin matrices as a delivery platform for urothelial cells to treat a canine model of urethral defect [226]. Microstructural studies showed that the electrospun matrices had a porous structure and supported the urothelial cells adhesion and proliferation. Retrograde urethrography showed no sign of US in the animals treated with the cell-seeded matrices. Furthermore, histopathological examinations showed that stratified epithelial layers were developed in the cell-matrix group. The healing activity of this delivery system may be attributed to the uniform urothelial cell layer on the matrices that may have prevented urinary extravasation and fibrotic reactions.

Similarly, Xie et al. cultured autologous oral keratinocytes and fibroblasts on electrospun silk fibroin matrices to develop an artificial buccal mucosa [66]. The healing function of these constructs was then evaluated in a canine model of a 5 cm-long urethra mucosal defect. Results showed that the autologous cells stayed viable on the matrices and formed a multilayer epithelium. In vivo study showed that the animals treated with the artificial buccal mucosa voided without difficulty, and US did not occur. Although no adverse tissue reactions were reported in these studies, silk fibroin-based scaffolds may generate chronic immunological reactions that can lead to graft failure or fibrosis [227]. 

In vivo urethra tissue engineering has also been investigated. In this technique, the cell-free scaffold is implanted at the defect site, allowing the resident cells to migrate on the scaffolds and populate it over time [228]. The criteria for a bioactive cell delivery system must be also considered in designing these constructs. In this framework, Hu et al. used poly D,L-lactic-co-glycolic acid (PLGA) and PLGA/gelatin scaffolds to treat urethral defects in a canine model. In vitro studies showed that the urothelial cells could adhere and propagate on both scaffolds. However, in vivo studies showed varying degrees of US in the repaired urethras. Histopathological examinations showed that multilayered disintegrated urothelium formed at both ends of the tubes. In addition, many polymorphonuclear cells had been infiltrated under the epithelium. They concluded that this system is unsuitable for treating urethra defects [229].

In contrast with this study, Liu et al. reported quite the opposite results. They functionalized PLLA with gelatin to develop a delivery vehicle for epithelial and smooth muscle cells [79]. The scaffolds had a fibrous structure and their surface hydrophilicity improved by increasing the gelatin fraction. None of the scaffolds were toxic against the seeded cells, and supported their adhesion and proliferation. However, the 75:25 weight ratio of PLLA gelatin had the best performance in the cell adhesion studies. ICC staining showed successful expression of AE1/AE3 and α-SMA in cell-scaffold constructs. The scaffolds were implanted into a rabbit model of urethra defect. Results showed that the animals treated with PLLA/gelatin grafts had significantly higher urine flow rate and urethra diameter compared with PLLA-only scaffolds. Histopathological examinations showed that in the PLLA/gelatin group, smooth muscle cells and urethral epithelial cells regularly grew on the scaffolds and populated their surface. US occurred the PLLA-only scaffolds, because these scaffolds were not conductive for cell adhesion and growth. The PLLA/gelatin scaffolds may have served as a migration substrate for the resident epithelial and smooth muscle cells and augmented the urethra defect repair (For the figures, please see reference). 

Niu et al. developed a bioactive nanofibrous platform to recruit the anti-inflammatory M2 macrophages into the urethra injury site [230]. The scaffolds were produced by coaxial electrospinning of hyaluronic acid and collagen. In vitro studies showed that the nanofibrous scaffolds induced the polarization of macrophages into the M2 phenotype. The healing function of these scaffolds was investigated in a male puppy model with an urethral defect. In animals treated with hyaluronic acid/collagen scaffolds, M2 macrophages were recruited onto the scaffolds and promoted the neovascularization and proliferation of endogenous urothelial progenitor cells. In this framework, various chemotactic factors can be loaded into electrospun scaffolds to recruit pro-healing cells at the injury site. For instance, AMD-3100 has been shown to recruit circulatory stem cells at the site of wound healing [231].

## 10. Previous Applications of Drug-Loaded Electrospun Delivery Systems to Treat Urethral Defects

**Figure 10:**
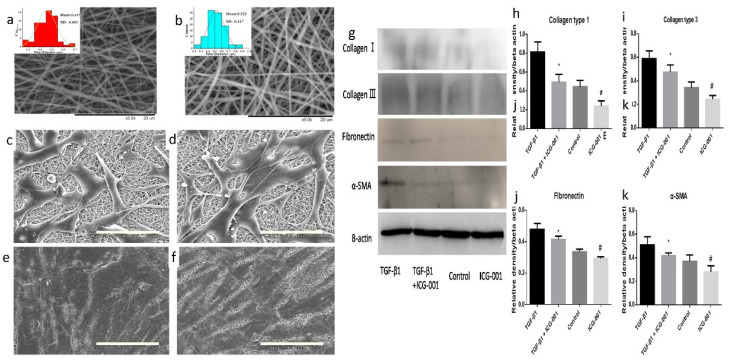
(**a**,**b**) show the microarchitecture of ICG-001-loaded and ICG-001-free scaffolds. (**c**,**e**) show the adhesion of epithelial cells on collagen/PLLA-CL scaffolds on days 3 and 7. (**d**,**f**) show the adhesion of epithelial cells on collagen/PLLA-CL/ICG-001 scaffolds on days 3 and 7. (**g**–**k**) show the Western blotting images of different proteins in fibroblast cells treated with TGFβ-1 and medium released from ICG-001/scaffolds. * and # show *p*-value < 0.05. Adopted from reference [232]. (**c**–**f**) Scale bars, 100 μm.

**Figure 11:**
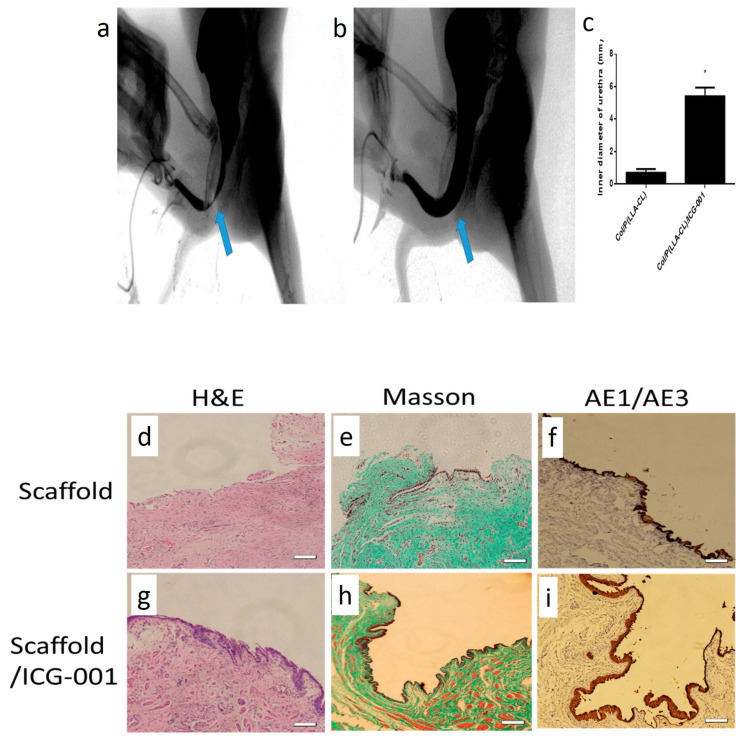
(**a**–**c**) show the urethrography images of animals treated with collagen/PLLA-CL and collagen/PLLA-CL/ICG-001 scaffolds, respectively. The blue arrow indicates the surgery position. (**d**–**i**) shows the histopathological examinations of urethra tissues in different groups. * shows *p*-value < 0.05. Adopted from reference [232]. Scale bars: 100 μm.

Electrospun scaffolds can serve as a drug delivery system and scaffolding platforms in urethra defects repair. In this context, anti-fibrotic drugs delivery via electrospun scaffolds has gained significant attention. For example, Zhang et al. loaded a Wnt signaling pathway inhibitor, ICG-001, into collagen/poly(L-lactide-co-caprolactone) (P(LLA-CL)) scaffolds using a coaxial electrospinning method for treating US [232]. The produced scaffolds had a fibrous structure that supported bladder epithelial cells adhesion and proliferation (Figure 10a–f). Fibroblast cells cultured with the extract of ICG-001-loaded scaffolds had significantly lower expression levels of collagen type 1 and 3, and fibronectin genes (Figure 10g–k). The healing function of this system was evaluated in a rabbit model of urethra defect. The study revealed that US and fistula in ICG-001-loaded scaffolds were significantly lower than in ICG-001-free groups (Figure 11a–c). Histopathological examinations showed a negligible collagen deposition, a thick epithelial, and smooth muscular layer in rabbits treated with ICG-001-loaded scaffolds (Figure 11d–i).

In another study, Zhang et al. investigated the therapeutic function of collagen/PLLA-CL/ICG-001 scaffolds in a canine model of urethral defect. Results were consistent with their previous work on the rabbit model, and ICG-01-delivering scaffolds prevented US and resulted in a fully functional urethra after 12 weeks of follow-up [233]. In another study, Guo et al. produced collagen/PLLA-CL/ICG-001 scaffolds. They combined the conjugated electrospinning and dynamic liquid electrospinning methods to fabricate the yarns. Their results showed that the cells cultured on the electrospun scaffolds had an organized morphology and penetrated into the constructs [234]. Although this study provided valuable information regarding the interaction of fibroblast cells with collagen/PLLA-CL/ICG-001 scaffolds, the lack of in vivo study is the main shortcoming of this research. In addition to ICG-001, PRI-724 that is an anti-fibrotic drug to treat liver cirrhosis and its active metabolite known as C-82 can potentially prevent US [235]. Therefore, incorporating these drugs into the electrospun scaffolds may increase their anti-fibrotic potential.

**Figure 12:**
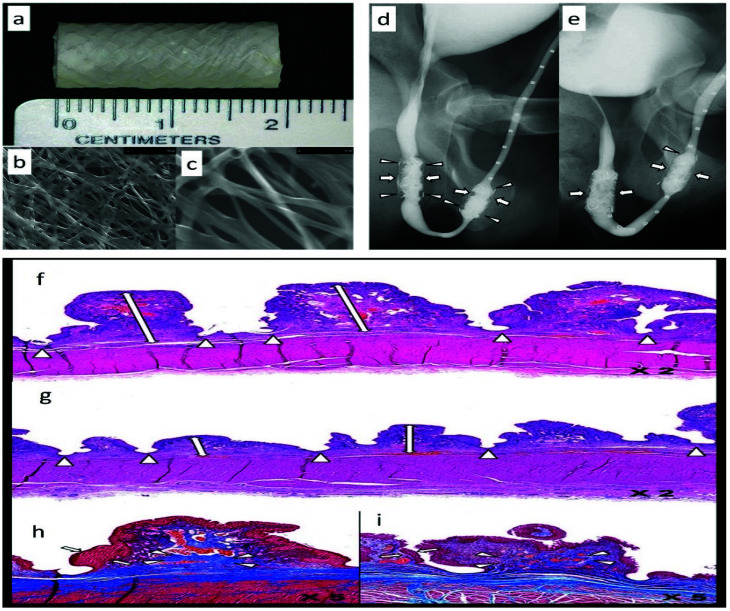
(**a**) shows the nanofiber-coated metallic stent, (**b**,**c**) show the microstructure of EW-7197 loaded nanofibers. (**d**,**e**) show the retrograde urethrography images of the drug-free and drug-loaded stent groups. (**f**,**g**) show the H & E staining images of the drug-free and drug-loaded stent groups. (**h**,**i**) show Masson’s trichrome staining images of the drug-free and drug-loaded stent groups. Adopted from reference [85].

TGF-β1 signaling plays a fundamental role in establishing and progression of fibrotic reactions. Therefore, many efforts have been dedicated to developing anti-fibrotic treatment modalities based on inhibiting TGF-β1 signaling [236]. EW-7197 is a TGF-β1 receptor kinase inhibitor that can be used to treat US [237]. In this regard, Han et al. loaded EW-7197 into PDLLA/PU electrospun fibers to develop an EW-7197-eluting nanofiber-covered stent. They studied its anti-US activity in a canine model of urethra defect [85]. The fibers deposited on the metallic stent, had a web-like architecture (Figure 12a–c). The urethrographic findings showed that at 4 and 8 weeks post-surgery, the luminal diameter of the urethra in drug-loaded stents was significantly larger than in drug-free stents (Figure 12d,e). Furthermore, histopathological examinations showed that the mean thickness of submucosal fibrosis and infiltration of inflammatory cells in the drug-loaded stent group were significantly lower than in the drug-free stent group (Figure 12f–i). This preliminary research suggests the potential applicability of EW-7197-delivering nanofibrous scaffolds to treat US in the clinic. Other TGF-β type-1 receptor kinase inhibitors, such as IN-1233, may also be of therapeutic value in treating US [238].

Small inhibitory RNA (siRNA) technology can knock down the genes involved in the fibrotic reactions [239]. For example, Xu et al. seeded oral keratinocytes and TGF-β1 siRNA transfected fibroblasts on electrospun PCL/silk fibroin/collagen matrices to treat US by blocking the TGF-β1 signaling pathway [240]. In vitro studies showed that the electrospun scaffolds were compatible with the seeded cells. Furthermore, in vivo study in a rabbit model revealed that the animals treated with the siRNA-delivering constructs had a wide urethra caliber. In addition, a stratified epithelial layer was formed in this group. Therefore, silencing pro-fibrosis genes via siRNA technology is a feasible strategy to prevent US after urethroplasty. However, siRNA is a hydrophilic molecule and cannot efficiently pass through hydrophobic plasma membranes. Furthermore, other challenges such as degradation by endonucleases, phagocytosis by the reticuloendothelial system, off-target effects, and activation of the immune responses are other challenges of siRNA technology in medical applications [241]. 

The chemokine stromal cell-derived factor-1 alpha (SDF-1α) and its ligand, the C-X-C chemokine receptor type 4 (CXCR-4), trigger the recruitment of resident and circulatory MSCs into the tissue injury site. The upregulation of SDF-1α after the tissue injury induces the infiltration of bone marrow-derived MSCs into the injured tissue [242,243]. Therefore, incorporating this agent into the electrospun scaffolds may induce homing of MSCs at the urethra injury and augment tissue healing. Liu et al. loaded SDF-1α into electrospun silk fibroin microfibers and deposited them onto the bladder acellular matrix grafts [244]. The healing activity of the produced constructs was then investigated in a rabbit model of ventral urethra defect. In vitro studies showed that SDF-1α-loaded scaffolds significantly promoted the migration of MSCs in the transwell assay. The animals treated with SDF-1α-loaded composite scaffolds showed optimal tissue regeneration and the least incidence of US or fistula. Histopathological examinations showed that a typical transitional epithelium with multiple layers was formed in SDF-1α-loaded composite scaffolds. Furthermore, Masson’s trichrome staining showed reduced fibrosis in SDF-1α-loaded composite scaffolds compared with other groups. This study indicated the beneficial role of MSC-recruiting drugs in treating urethral defects. One can develop a dual-function delivery system for these drugs and MSCs. In this strategy, the secretome of the recruited MSCs and seeded MSCs act in a synergistic manner and promote tissue repair [231]. 

## 11. Challenges and Potential Mitigation Strategies

Despite considerable progress, urethra defect repair using electrospun cell and drug delivery systems is still in its infancy and faces many limitations and challenges. Considering the solvent systems used for developing the electrospun cell and drug delivery systems, most studies have utilized class 2 solvents that may potentially elicit adverse tissue reactions and cytotoxicity [8]. The easy optimization of electrospinning parameters with these solvents may reason this crucial point to be neglected. The concept of “Green electrospinning” has been proposed to address this issue. This technology uses less toxic solvent systems to produce electrospun scaffolds. Furthermore, drying the electrospun scaffolds in a vacuum chamber may also extract the residual solvents from the matrix of electrospun scaffolds [245]. 

In the case of natural polymers-based electrospun scaffolds, fast biodegradation rate and poor mechanical strength necessitate cross-linking these constructs before implantation [246]. While glutaraldehyde is a widely used reagent for cross-linking, its potential cytotoxicity may compromise the cell viability in electrospun cell delivery systems. Keeping this limitation in mind, green cross-linking reagents with minimal toxic effects have been developed [247]. Furthermore, natural polymers show batch-to-batch variations in physicochemical and biological properties. This challenge can lead to therapeutic outcome discrepancies with different sources [248]. 

Given the dose-dependent effects of anti-fibrotic drugs, spatiotemporal control over these drugs release is an essential requirement of drug-loaded electrospun scaffolds [249]. In vitro drug release assays can partly simulate the drug release rate from polymeric scaffolds. However, the interplay between various factors such as reactive oxygen species (ROS), the secretome of inflammatory cells, ions, and enzymes may significantly change the drug release profile from these constructs [93]. 

Concerning the clinical translation, the effects of sterilization on mechanical properties and microstructure of electrospun cell and drug delivery systems must be kept in mind. For example, the current sterilization methods may alter the surface topography on the electrospun scaffolds, resulting in different cellular behavior in electrospun cell delivery systems [250,251]. Furthermore, acquiring FDA approval for clinical translation of different cell types will be demanding. Although no adverse effects have been reported in preclinical studies, clinical data regarding these cells safe application in human subjects is lacking. 

Converting electrospun cell and drug delivery systems from R&D to mass production scale is also challenging. The low production yield of the conventional electrospinning method does not meet the criteria of a high throughput manufacturing technique. Therefore, recent studies have proposed increasing the number of fiber jets to improve the production yield [252,253]. 

Finally, the natural healing response in the urethra defect involves the interplay between various cells and cues [53]. Therefore, the electrospun cell or drug delivery systems should accommodate multiple cells or bioactive agents to recapitulate this environment. However, mimicking this complex network is beyond the capability of the existing technology. 

## 12. Conclusions and Future Perspectives

The electrospun scaffolds are versatile platforms for cell or drug delivery applications to treat urethra defects. The inherent resemblance of these constructs to native cell niches and their modifiable physicochemical and biological properties have led to their widespread applications in cell delivery. In the context of urethral defect repair, cells from various sources such as stem cells, buccal mucosal cells, urothelial cells, and smooth muscle cells have been delivered via these scaffolds to augment tissue repair. Furthermore, high surface-to-volume ratio, high encapsulation efficacy, and tunable drug release are other advantages of these constructs to deliver bioactive agents. In this framework, various anti-fibrosis drugs have been delivered via electrospun scaffolds to prevent US recurrence or its development. These scaffolds have also been used as a dual-function delivery system for both cells and bioactive agents to target different factors involved in the pathophysiology of US. Despite these endeavors, the current state-of-the-art falls far behind the clinical translation stage. Many of the challenges, such as the complexity of innate cell niches, spatiotemporal control over drug release, the batch-to-batch variations of natural polymers, the scale-up issues, the production challenges with safe solvent systems, the adverse effects of sterilization techniques on electrospun scaffolds, and the difficulties in acquiring regulatory approvals, need to be addressed before any clinical trial can take place. 

Concerning future research, the anti-US function of various drugs, such as corticosteroids, angiotensin-converting enzyme inhibitors, and different inhibitors of fibrosis-associated signaling pathways in an electrospun formulation, has not been investigated. Furthermore, the fibrotic reactions following urinary extravasation may occur by orchestrated activation of various signaling pathways, including Wnt/β-catenin, YAP/TAZ, and TGF-β signaling systems. Therefore, blocking all these pathways simultaneously may result in better anti-US outcomes. Finally, mimicking the natural cell niches using advanced drug delivery systems and providing biological cues may also unleash the potential of cell-seeded electrospun scaffolds to treat urethra defects.

## Figures and Tables

**Table 2:** Parameters affecting the properties of electrospun fibers.

Parameter	Effects	References
The molecular mass of the polymer	Polymers with low molecular mass tend to form beads, while higher molecular mass results in fibers with a more uniform structure	[161]
Viscosity	The higher molecular mass and polymer concentration increase the viscosity of the polymeric solution. As a result, viscose solutions tend to produce thicker fibers	[162]
Surface tension	Increasing the polymer concentration reduces the surface tension and leads to fibers with a continuous and uniform structure. Changing the solvent system or adding surfactant can also alter surface tension	[163]
Conductivity	The polymeric solution’s conductivity affects the fibers’ mean fiber size and morphology. Incorporation of salts or polyelectrolytes can improve conductivity	[164]
Applied voltage	High voltages decrease the average fiber diameter. Furthermore, high voltages stretch the fibers and align the polymeric chains, increasing fibers’ crystallinity	[165]
Solution flow rate	The average fiber diameter will increase with higher flow rates and vice versa	[166]
Needle to collector distance	The increase in the fiber receiving distance to a certain extent will produce ultrafine fibers. Therefore, the distance should be optimized for every polymeric solution; otherwise, disintegrated or beady fibers will be produced	[167]
Properties of the receiver	The architecture and morphology of the fibers can be determined by using different collectors. Increasing the collector’s rotation rate increases the alignment of fibers. In addition, 3D electrospun matrices can be produced when fibers are spun into a liquid coagulation bath	[168,169]
Humidity	Humidity can alter the solvent’s humidity and cause fibers’ fusion	[170]
Temperature	Temperature can alter viscosity, solvents’ volatility, and surface tension. In addition, high-temperature results in rapid solvent evaporation and reduces the flying time, thereby increasing fibers’ diameter	[171]
Air pressure	The air pressure affects the solvent’s volatility and jet stability. However, fibers with a uniform structure and consistency can be produced when spun at a constant air pressure	[172]

## Data Availability

No data was generated in this study.
